# Conformational Properties of the Unfolded State of Im7 in Nondenaturing Conditions

**DOI:** 10.1016/j.jmb.2011.12.041

**Published:** 2012-02-17

**Authors:** Clare L. Pashley, Gareth J. Morgan, Arnout P. Kalverda, Gary S. Thompson, Colin Kleanthous, Sheena E. Radford

**Affiliations:** 1 Astbury Centre for Structural Molecular Biology, University of Leeds, Leeds LS2 9JT, UK; 2 Institute of Molecular and Cellular Biology, University of Leeds, Leeds LS2 9JT, UK; 3 Department of Biology, University of York, York YO10 5YW, UK

**Keywords:** TS1, transition state 1, TS2, transition state 2, ColE7, colicin E7, SSP, secondary structure propensity, smFRET, single-molecule Förster resonance energy transfer, Im7, immunity protein 7, EDTA, ethylenediaminetetraacetic acid, HSQC, heteronuclear single quantum coherence, AUC, analytical ultracentrifugation, ITC, isothermal titration calorimetry, BMRB, Biological Magnetic Resonance Data Bank, NOE, nuclear Overhauser enhancement, AABUF, average area buried upon folding, PDB, Protein Data Bank, protein folding, NMR, unfolded ensemble, denatured state, immunity protein

## Abstract

The unfolded ensemble in aqueous solution represents the starting point of protein folding. Characterisation of this species is often difficult since the native state is usually predominantly populated at equilibrium. Previous work has shown that the four-helix protein, Im7 (immunity protein 7), folds via an on-pathway intermediate. While the transition states and folding intermediate have been characterised in atomistic detail, knowledge of the unfolded ensemble under the same ambient conditions remained sparse. Here, we introduce destabilising amino acid substitutions into the sequence of Im7, such that the unfolded state becomes predominantly populated at equilibrium in the absence of denaturant. Using far- and near-UV CD, fluorescence, urea titration and heteronuclear NMR experiments, we show that three amino acid substitutions (L18A–L19A–L37A) are sufficient to prevent Im7 folding, such that the unfolded state is predominantly populated at equilibrium. Using measurement of chemical shifts, ^15^N transverse relaxation rates and sedimentation coefficients, we show that the unfolded species of L18A–L19A–L37A deviates significantly from random-coil behaviour. Specifically, we demonstrate that this unfolded species is compact (*R*_h_ = 25 Å) relative to the urea-denatured state (*R*_h_ ≥ 30 Å) and contains local clusters of hydrophobic residues in regions that correspond to the four helices in the native state. Despite these interactions, there is no evidence for long-range stabilising tertiary interactions or persistent helical structure. The results reveal an unfolded ensemble that is conformationally restricted in regions of the polypeptide chain that ultimately form helices I, II and IV in the native state.

## Graphical Abstract

**Figure:**
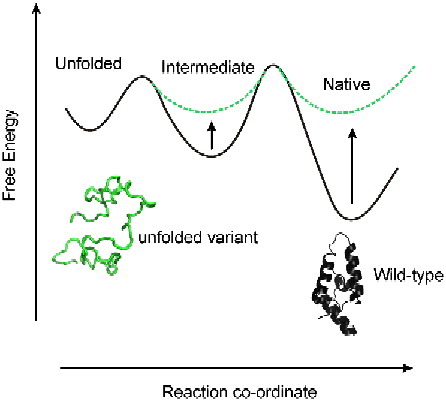

**Highlights:**

► An unfolded variant of Im7 has been created in nondenaturing conditions. ► The variant is collapsed but lacks fixed secondary or tertiary structure. ► Transient helicity and collapse prime the protein towards folding.

## Introduction

Characterisation of all species formed during the folding process is required to understand how a protein folds in atomistic detail.^1^ Conformations that are transiently sampled within the denatured ensemble represent an interesting, but experimentally challenging, starting point of folding. The unfolded state may dictate the conformations open to a protein during its lifetime by restricting the conformational space available to the polypeptide chain. Accordingly, the presence of residual structure in the unfolded state has been suggested as one property that could help resolve Levinthal's long-standing paradox.^1,2^ Assuming folding takes place on a funnelled landscape,^3^ residual structure could reduce the conformational space available to the unfolded protein chain at the commencement of folding, potentially reducing the time spent searching for the native conformation. The general consensus from many studies is that residual structure in the unfolded state could play an important role in protein folding.^4–7^ Residual structure may provide initiation points for folding,^5,8^ affect the stability of the native protein^9^ and/or modulate folding mechanisms and rates.^4^ These findings highlight the importance of delineating the structural, thermodynamic and kinetic properties of the unfolded state.

Assessing the nature of residual structure in the unfolded state of a natively folded protein is experimentally challenging since this species is rarely populated at equilibrium under conditions favouring folding. There are proteins that naturally lend themselves to the study of the unfolded state (i.e., using naturally unstable proteins or proteins requiring a cofactor to fold).^10,11^ However, steps are usually required to increase the population of unfolded conformers under ambient conditions before biophysical studies can be undertaken. A number of different approaches have been utilised, including unfolding the protein at low pH, denaturation at low or high temperature or the addition of chaotrope.^12–17^ Alternatively, mutagenesis, sometimes in combination with methionine oxidation, has been used to increase the population of the unfolded state at equilibrium.^18–21^ Extrapolation to low denaturant concentrations has also been carried out using single-molecule Förster resonance energy transfer (smFRET) experiments.^22–24^ The results of these studies revealed that nonrandom behaviour can persist in denatured states of proteins, even under harsh denaturing conditions. These results provide compelling evidence that unfolded species under ambient conditions also possess significant residual native or nonnative structure, albeit that these structures may be rare or transient. Since this is the starting point for protein folding *in vitro*, understanding the structural properties of the unfolded ensemble under nondenaturing conditions is fundamental to our understanding of the folding process.

**Fig. 1:**
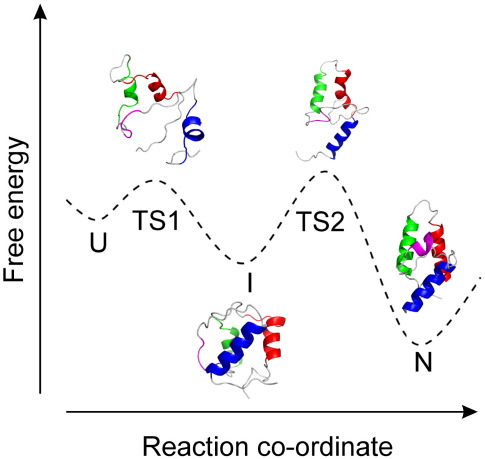
Schematic representation of the folding energy landscape of Im7. Species populated during folding are indicated as follows: U, unfolded state; I, intermediate state; N, native state; TS1, the first transition state; TS2, the rate-determining transition state. The structure of Im7 (PDB code: 1AYI^33^) is shown for the native state, and representative structures from the ensemble generated by restrained molecular dynamics simulations are shown for TS1, I and TS2.^27,34^ Regions that form the four helices in the native state are coloured red (helix 1), green (helix 2), pink (helix 3) and blue (helix 4).

The folding mechanism of the colicin immunity protein 7 (Im7) has been extensively characterised as a model for helical protein folding.^24–30^ Despite its small size, this 87-residue protein has been shown to fold to its native state *via* an on-pathway intermediate (I), stabilised by both native and nonnative interactions.^31,32^ Using a combination of protein engineering (Φ-value analysis), hydrogen exchange protection experiments and restrained molecular dynamics simulations, it has been possible to obtain all-atom models of the ensembles for the early and late transition states (TS1 and TS2) and the populated intermediate (I), allowing an atomic model of the Im7 folding mechanism (Fig. 1).^27^ These experiments revealed that TS1 is expanded (β_T_^TS1^ = 0.24), where the β-Tanford value (β_T_) is an indirect measure of the average solvent-exposed surface area in a given state relative to that of the denatured and native states. β_T_^*x*^ therefore indirectly reflects the overall compactness of the species, *x*.^35^ If β_T_^*x*^ = 0, the species, *x*, has the same average solvent-exposed surface area as the denatured state, whereas if β_T_^*x*^ = 1, the species has an average solvent-exposed surface area similar to that of the native state.^35^ Furthermore, Φ-value analysis indicated that TS1 lacks ordered secondary structure, although there is evidence for persistent long-range interactions between helices I and II in this species.^27^ The transition from TS1 to the more ordered, on-pathway intermediate (I) is a key step in the folding of Im7 and involves substantial compaction (β_T_^I^ = 0.74); the formation of stable native-like secondary structure in helices I, II and IV; desolvation of the core; helix–helix docking; and the formation of both native-like and nonnative stabilising interactions.^27^ The rate-limiting step occurs as the protein traverses TS2 from I to form the native state.^27^ Subtle rearrangements of the helices and core along with further compaction (β_T_^TS2^ = 0.89) take place in TS2, enabling formation of the binding site for helix III. Helix III then forms and docks in the last step of folding (Fig. 1).^27^

Despite the extensive knowledge of the folding mechanism of Im7, our understanding of the starting point of folding (i.e., the unfolded state under nondenaturing conditions) remained limited. The urea-denatured state of Im7 has been characterised in 6 M urea and was shown to be highly expanded and to lack residual secondary structure or stable long-range interactions.^29^ However, recent smFRET studies of Im7 suggested that the unfolded state in the absence of denaturant is significantly more compact than the urea-denatured state in 6 M urea in that the FRET efficiency between the donor and acceptor dyes (i.e., the proximity ratio) of the former approaches that of the native state in 0 M urea.^24^ Here, protein engineering has been used to trap Im7 in an unfolded state in the absence of denaturant, enabling determination of its structural properties using fluorescence, CD spectroscopy and heteronuclear NMR methods under the solution conditions used to monitor folding (pH 7, 10 °C).^24,26–28^ The results reveal a collapsed ensemble that lacks persistent helical structure. Folding of Im7 may then progress by the consolidation of the collapsed regions to form cooperative stabilising interactions that promote secondary- and tertiary-structure formation once folding commences.

## Results

### Design of an unfolded Im7 variant

**Fig. 2:**
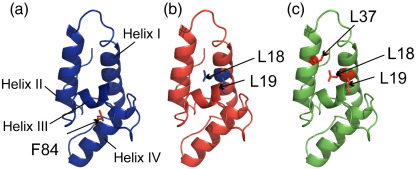
Ribbon diagrams of Im7 (PDB code: 1AYI^33^) showing its four native helices. The location of residues mutated in the variants (a) F84A, (b) L18A–L19A and (c) L18A–L19A–L37A are indicated. The figure was created using PyMOL.

Wild-type Im7 has a folding free energy (Δ*G*°_UN_) of − 24.9 kJ mol^− 1^ at 10 °C, pH 7.0, in 0.4 M Na_2_SO_4_, the conditions that have been used to study its folding mechanism.^24,26–28^ Under these conditions, the partially folded intermediate is also stable (Δ*G*°_UI_ = − 11.9 kJ mol^− 1^).^30^ Significant destabilisation of both the native and intermediate states is required to populate the unfolded state at equilibrium in the absence of denaturant. Amino acid substitutions to the sequence of Im7 made previously during Φ-value analysis were considered to achieve this.^27,30^ The aim was to combine one or more of these substitutions to destabilise the native and intermediate states of the protein, such that these species are no longer significantly populated at pH 7.0 and 10 °C. Previous Φ-value analysis demonstrated that the amino acid substitution F84A is highly destabilising, such that the folding kinetics of this variant could not be measured.^30^ Substitution of this amino acid was considered desirable since this residue is buried in both the intermediate and native states but is not involved in native helical structure (Fig. 2a).^27,33^

Since substitution of a large aromatic group could potentially affect the residual structure in the unfolded state by reducing hydrophobic collapse, the more subtle substitutions L18A, L19A and L37A in helices I and II were also considered. These sequence alterations have each been shown to destabilise both N and I significantly (Δ*G*°_UN_ of − 12.6, − 14.2 and − 11.9 kJ mol^− 1^ and Δ*G*°_UI_ = − 3.7, − 5.8 and − 4.3 kJ mol^− 1^, respectively).^30^ Previous Φ-value analysis reveals Φ_TS1_ values of 0.31 ± 0.19, 0.38 ± 0.19 and 0.28 ± 0.15; Φ_I_ values of 0.45 ± 0.09, 0.56 ± 0.09 and 0.28 ± 0.11; and Φ_TS2_ values of 0.67 ± 0.42, 0.70 ± 0.41 and 0.53 ± 0.18, for the individual L18A, L19A and L37A amino acid substitutions, respectively.^30^ A double-substitution variant L18A–L19A, along with a triple-substitution variant L18A–L19A–L37A, was created in order to try to populate the unfolded state predominantly at equilibrium in the absence of denaturant (Fig. 2b and c).^30^ Residues involved in the binding interface between Im7 and its cognate binding partner colicin E7 (ColE7) were not altered in any design.^36^ The Im7–E7 complex has a Δ*G*°_binding_ of − 83.2 kJ mol^− 1^.^36^ As a consequence, the ability of the variants to undergo binding-induced folding could be determined as a test of the ability of the variants to reach a functional state despite the amino acid substitutions introduced.^37^ The Im7 variants F84A, L18A–L19A and L18A–L19A–L37A were expressed in *Escherichia coli* and purified to homogeneity as described previously (Materials and Methods).^38^ Their structural characteristics, unfolding properties and stability were then deduced using a range of biophysical methods.

### Structural properties of the Im7 variants

**Fig. 3:**
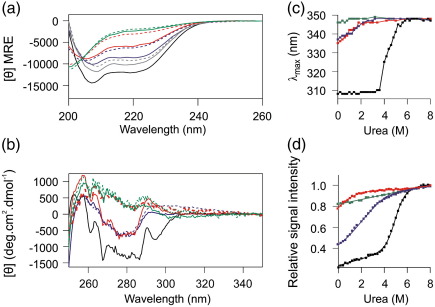
Summary of CD and fluorescence spectroscopy of the Im7 variants in buffer A at 10 °C. (a) Far-UV CD spectra of the different variants of Im7 at 10 °C. Dotted lines indicate spectra in 0 M Na_2_SO_4_ and a continuous line indicates spectra in the presence of 0.4 M Na_2_SO_4_. Spectra of wild-type Im7, which is independent of Na_2_SO_4_ (black), F84A (blue), L18A–L19A (red) and L18A–L19A–L37A (green), and the trapped intermediate state I53A L54A (grey) are shown.^32^ These colours are used to indicate the different variants in all panels. The units of mean residue ellipticity (MRE) are deg cm^2^ dmol^− 1^ peptide bond^− 1^. (b) Near-UV CD spectra of the different variants in the presence of 0 M Na_2_SO_4_ (dotted line) or 0.4 M Na_2_SO_4_. The spectrum of wild-type Im7 in 0.4 M Na_2_SO_4_ is shown in black. Urea-induced denaturation of wild-type Im7 and the variants measured (c) by the change in fluorescence emission λ_max_ and (d) by the change in the CD signal at 222 nm in the presence of 0.4 M Na_2_SO_4_. CD data are normalised to the signal in 8 M urea before conversion to signal intensity relative to this value. Data for the wild-type protein (black) are fitted to a model describing a two-state transition [Eq. (2)], as described in Materials and Methods. All other data were fitted using a straight line, or polynomial function, to guide the eye.

A series of experiments were performed in order to determine which, if any, of the variants created are unfolded under ambient conditions as a prelude to their more detailed structural characterisation using NMR. First, CD spectroscopy was used to assess the secondary and tertiary structure of each variant. Previous protein folding studies of Im7 were performed in 50 mM sodium phosphate (pH 7.0) containing 1 mM ethylenediaminetetraacetic acid (EDTA) (buffer A) and 0.4 M Na_2_SO_4_ at 10 °C. Since Na_2_SO_4_ is known to stabilise the intermediate and native states of Im7, but not to alter their structural properties,^39^ CD spectra were recorded in the presence and absence of this kosmotrope (Fig. 3). Wild-type Im7 has a helical content of 50%, which does not change upon the addition of Na_2_SO_4_.^33,39^ The far-UV CD spectrum of this protein was used to calculate the helical content of each of the variant proteins relative to that of the native state (Materials and Methods).^32,33^ The far-UV CD spectrum of F84A shows that this variant contains significant helicity (∼ 25%) (Fig. 3a). Near-UV CD showed that this protein also retains fixed tertiary structure, although this differs from that of the native protein (Fig. 3b). The helical content of F84A increases slightly upon the addition of 0.4 M Na_2_SO_4_ (∼ 36% helicity) (Fig. 3a), while there is little effect of Na_2_SO_4_ on the tertiary structure as indicated by near-UV CD (Fig. 3b). The L18A–L19A variant behaves differently. In the absence of Na_2_SO_4_, this variant has little helical content (∼ 12%) and no detectable fixed tertiary structure (Fig. 3a and b). However, in the presence of 0.4 M Na_2_SO_4_, the helical content of L18A–L19A increases to ∼ 25% and the near-UV CD spectrum indicates that this variant adopts fixed, nonnative tertiary interactions (Fig. 3b). By contrast, L18A–L19A–L37A has little helical structure (∼ 9%) and lacks fixed tertiary interactions involving aromatic side chains, independent of the concentration of Na_2_SO_4_ (Fig. 3a and b).

Superposition of the far-UV CD spectra of the variants (Fig. 3a) reveals an isodichroic point that is not shared by the wild-type protein. This suggests that the variants are in equilibrium between two or more conformations with distinct secondary structure, irrespective of the amino acid substitutions introduced. Significantly, the double mutant L53A I54A, which has previously been shown to populate the I-state to greater than 90% in 0.4 M Na_2_SO_4_,^32^ shares the isodichroic point of the variants (Fig. 3a). This suggests that the variants created are in equilibrium between the I and U states, with the population of each state being dependent on the concentration of Na_2_SO_4_ and the amino acid substitutions introduced.

A cooperative unfolding transition is a key characteristic of natively folded proteins.^40^ Accordingly, the absence of a cooperative unfolding transition upon the addition of urea was taken here as an additional criterion for population of an unfolded species. Urea titrations of each variant were performed using far-UV CD (θ_222 nm_) and fluorescence emission (λ_max_) of the single tryptophan (W75) as structural probes (Fig. 3c and d). The data show that in the presence of 0.4 M Na_2_SO_4_, F84A and L18A–L19A exhibit evidence of a nonlinear dependence of the CD and fluorescence signal on the denaturant concentration, indicative of an unfolding transition. However, L18A–L19A–L37A lacks evidence of significant nonlinearity in the urea titrations, demonstrating the absence of a detectable cooperative unfolding event (Fig. 3c and d). Consistent with the far- and near-UV CD spectra, in the absence of Na_2_SO_4_, F84A and L18A–L19A appeared less stable than their Na_2_SO_4_-stabilised counterparts (data not shown). Interestingly, even though L18A–L19A–L37A lacks evidence of cooperative unfolding, there is a  2-nm blue shift in the λ_max_ of this variant in 0 M urea compared with 8 M urea in the presence of 0.4 M Na_2_SO_4_ (Fig. 3c). This shift is not observed in the absence of Na_2_SO_4_ (data not shown), consistent with Na_2_SO_4_ inducing collapse of this highly destabilised variant.

### Sodium sulfate perturbs the unfolded ensemble

**Fig. 4:**
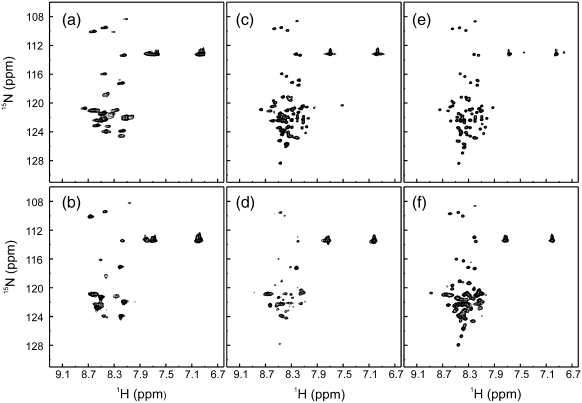
^1^H–^15^N HSQC spectra (500 MHz) of Im7 variants. Spectra of F84A (a and b), L18A–L19A (c and d) and L18A–L19A–L37A (e and f) were acquired in buffer A (90% ^1^H_2_O/10% ^2^H_2_O) with 0 M Na_2_SO_4_ (a, c and e) or 0.4 M Na_2_SO_4_ (b, d and f) at 10 °C.

The data presented above suggest that the variants studied here either populate the unfolded state predominantly at equilibrium (L18A–L19A–L37A) or exhibit conformational exchange between the intermediate and the unfolded state (L18A–L19A and F84A). To assess the feasibility of characterising the unfolded ensemble of these proteins in more detail using NMR, we acquired the ^1^H–^15^N heteronuclear single quantum coherence (HSQC) spectrum of each variant in 0 M urea and 0 M or 0.4 M Na_2_SO_4_ at 10 °C (Fig. 4). The spectra of F84A show significant line broadening both in the presence and in the absence of Na_2_SO_4_, consistent with conformational exchange between different species occurring on the microsecond-to-millisecond timescale (Fig. 4a and b). The ^1^H–^15^N HSQC spectrum of L18A–L19A in the absence of Na_2_SO_4_ (Fig. 4c and d) shows sharp resonances and limited dispersion in the ^1^H dimension, consistent with this variant predominantly populating the unfolded state in the absence of Na_2_SO_4_ (Fig. 4c). However, significant line-broadening effects are observed for L18A–L19A in 0.4 M Na_2_SO_4_, in accord with the results presented in Fig. 3. Finally, the ^1^H–^15^N HSQC spectrum of L18A–L19A–L37A in the absence of Na_2_SO_4_ shows limited dispersion in the ^1^H dimension and sharp resonances, characteristic of an unfolded protein (Fig. 4e). Upon the addition of 0.4 M Na_2_SO_4_, the ^1^H–^15^N HSQC spectrum of L18A–L19A–L37A exhibits line broadening compared with the spectrum in 0 M Na_2_SO_4_ (Fig. 4f). This is likely caused by an additional contribution to the transverse relaxation rate caused by conformational exchange (i.e., U-to-I equilibrium) that is slow–intermediate exchange on the chemical shift timescale. The presence of Na_2_SO_4_ may increase the population of the minor species in equilibrium with the unfolded state and/or alter the rate of exchange between species causing the line broadening observed in Fig. 4f. Overall, the L18A-L19A-L37A variant populates the unfolded state to the greatest extent. This variant was taken forward for more detailed structural analysis.

### The unfolded state is more compact than the urea-denatured state

Compaction of the unfolded state of Im7 in the absence of denaturant has been observed previously using single-molecule experiments.^24^ Compaction has also been observed in single-molecule experiments for many, although not all proteins.^41^ Other proteins for which smFRET studies have reported collapse of the unfolded state as denaturant is diluted include RNaseH,^42^ immunity protein 9,^43^ cold shock protein Csp*Tm*,^44^ chymotrypsin inhibitor,^45^ acyl-co-enzyme binding protein^45^ and barstar*.*^46^ To obtain a quantitative measure of the extent of compaction of L18A–L19A–L37A, we performed sedimentation velocity analytical ultracentrifugation (AUC) experiments, from which an estimate of the radius of hydration (*R*_h_) in buffer A at 10 °C was made (Materials and Methods). The measured *R*_h_ value of the native wild-type protein is 17.8 ± 0.3 Å (Table 1), consistent with the value expected for a protein the size of Im7 using the empirical method of Wilkins *et al.* (17.7 Å).^47^ The *R*_h_ measured for the L18A–L19A–L37A variant in buffer A with 6 M urea is 34.0 ± 1.73 Å, which is larger than 29.4 Å expected for an unfolded chain of this length (Table 1).^47^ Nonetheless, these measurements are consistent with the urea-denatured state of Im7 being highly expanded, with dimensions that indicate that the protein is as extended as expected for a random coil.^47^ The *R*_h_ for L18A–L19A–L37A in the absence of urea in buffer A with 0 M or 0.4 M Na_2_SO_4_, determined using sedimentation velocity AUC, was 26.1 ± 0.6 Å and 24.6 ± 0.8 Å, respectively, consistent with these unfolded species being more compact than their urea-denatured counterparts. Minor differences in the *R*_h_ values of L18A–L19A–L37A in 0 M and 0.4 M Na_2_SO_4_ are within experimental error. In comparison to the folded protein, the *R*_h_ of L18A–L19A–L37A is approximately 1.4 times the *R*_h_ measured for wild-type Im7. The frictional ratio for each species also supports the finding that L18A–L19A–L37A is more compact in the absence of urea (Table 1). Overall, these data suggest that L18A–L19A–L37A is approximately 30% more compact than its urea-denatured counterpart.

### Binding of ColE7 induces the folding of unfolded Im7

**Fig. 5:**
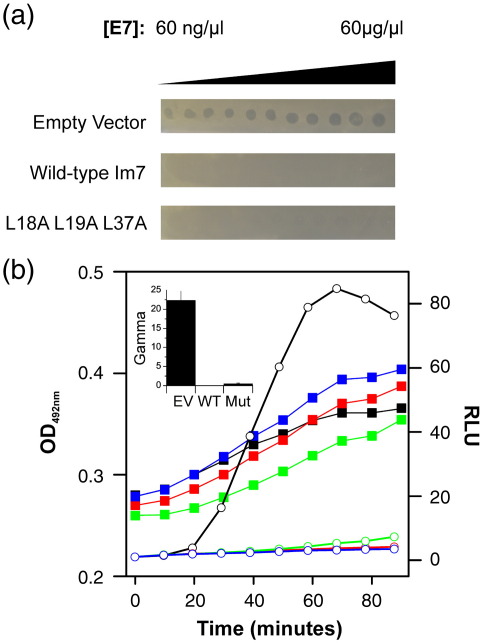
*In vivo* cell protection experiments to assay colicin activity. (a) ColE7 was spotted onto lawns of *E. coli* carrying the empty pTrc99A vector or the same vector containing the gene for wild-type Im7 or L18A–L19A–L37A. Protein production was induced by the addition of 1 mM IPTG prior to the addition of the ColE7. The dark spots indicate regions where colicin has killed the lawn of *E. coli*, indicating the lack of inhibition of ColE7. (b) Cell growth monitored at OD_492_ (filled squares) and relative luminescence units (RLU) (open circles) are shown for cultures of *E. coli* DPD1718 carrying the empty vector pTrc99A (black) or the same vector containing the genes for wild-type Im7 (red) or Im7 L18A–L19A–L37A (green) as a function of time after the addition of 4 nM ColE7. As a control, cells expressing wild-type Im7 were monitored without the addition of ColE7 (shown in blue). In all cases, 1 mM IPTG was used to induce expression of the Im7 gene 60 min prior to the addition of ColE7. Gamma values are shown in the inset and reflect the relative luminescence level induced by the addition of 4 nM ColE7 to cultures of *E. coli* DPD1718 carrying the empty vector pTrc99A (EV) or the same vector containing the gene for wild-type Im7 (WT) or the Im7 L18A–L19A–L37A variant (Mut). Gamma values were calculated as described in Materials and Methods. The error bars shown are the standard deviation from six repeat experiments.

While the experiments described above suggest that L18A–L19A–L37A is unfolded, it is important to verify that the engineered unfolded sequence is capable of folding to the native state provided that suitable stabilising conditions can be found. Binding studies with ColE7 were performed using *in vivo* protection assays against ColE7 toxicity to investigate the potential of this variant to fold *in vivo*.^48^ Since the amino acid substitutions introduced into the variants do not involve binding site residues,^49^ the free energy of binding for Im7–E7 (Δ*G*°_binding_ = − 83.2 kJ mol^− 1^ at 25 °C)^36^ should provide sufficient energy to fold the variant, provided that the sequence is compatible with a native structure. A lawn of *E. coli* overexpressing the Im7 variant of interest was grown in the presence of differing concentrations of ColE7 to determine if this is the case for L18A–L19A–L37A.^48^ When expression of wild-type Im7, or an Im7 variant, is induced by IPTG, cells survive only if Im7 is able to bind and inactivate the ColE7. Hence, zones of clearance are observed in this assay for cells carrying Im7 sequences incapable of folding and binding to ColE7. These experiments showed that expression of L18A–L19A–L37A in *E. coli* JM83 is able to provide protection against ColE7, consistent with binding-induced folding of L18A–L19A–L37A (Fig. 5a).

To further confirm the ability of L18A–L19A–L37A to provide protection against ColE7, we used a more sensitive, stress-induced reporter system (Materials and Methods) (Fig. 5b). This system utilises *E. coli* DPD1718, which has been engineered to contain a heterologous *luxCDABE* gene complex under the control of the cellular stress-induced *E. coli recA* promoter.^50^ The *luxA*, *luxB*, *luxC*, *luxD* and *luxE* genes express the structural proteins responsible for bacterial bioluminescence. Hence, luminescence can be detected when the *luxCDABE* genes are expressed under the control of a stress-induced promoter activated by colicin-induced DNA damage.^50^ To exploit this assay, we transformed *E. coli* DPD1718 cells with the pTrc99a vector containing the gene for L18A–L19A–L37A. Cells were grown to an OD_600_ (optical density at 600 nm) of 0.3, and protein expression was then induced by the addition of 1 mM IPTG for 1 h. The cells were then challenged by the addition of 4 nM ColE7, and luminescence, indicative of the induction of the stress response, was monitored (Fig. 5b). A “gamma value” was calculated to quantify colicin activity (Materials and Methods), with higher gamma values corresponding to a greater colicin activity within the cell.^50^ These experiments also indicated that L18A–L19A–L37A provides protection against ColE7, to a level similar to that provided by wild-type Im7 (Fig. 5b). These results are in accord with the results of the colicin protection assays (Fig. 5a) and confirm that L18A–L19A–L37A is able to fold to a functional entity capable of inactivating ColE7 upon their binding.

**Fig. 6:**
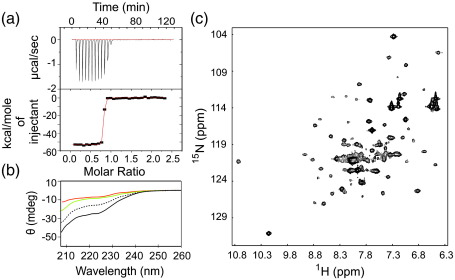
*In vitro* assays of Im7 binding to the ColE7 DNase domain. (a) ITC binding isotherm obtained by the titration of Im7 L18A–L19A–L37A into the DNase domain of ColE7 (upper panel); the red line in the lower panel shows a fit of the data to a 1:1 binding model as a guide to the eye only (see Materials and Methods for details). (b) Far-UV CD spectra taken in buffer A at 10 °C of 40 μM ColE7 E7 DNase domain alone (green), 10 μM L18A–L19A–L37A alone (red) and the spectrum of both proteins mixed together (black). The black dotted line represents the summed spectra of ColE7 and L18A–L19A–L37A, indicating the expected result if the proteins do not interact. (c) ^1^H–^15^N HSQC spectrum of a  500-μM solution of ^15^N-labelled L18A–L19A–L37A mixed 1:1 with ^14^N-labelled DNase domain of ColE7 at 10 °C in buffer A (90% ^1^H_2_O/10% ^2^H_2_O) with 0.2 M Na_2_SO_4_.

To confirm binding-induced folding of L18A–L19A–L37A, we also performed *in vitro* binding studies of this variant to the DNase domain of ColE7 using far-UV CD, ^1^H–^15^N NMR and isothermal titration calorimetry (ITC) (Fig. 6). Since Im7 binds only to the DNase domain of ColE7, the isolated ColE7 DNase domain was used for the *in vitro* binding experiments. ITC experiments (Fig. 6a) revealed that L18A–L19A–L37A binds to the DNase domain of ColE7 with a *K*_d_ in the subnanomolar range.^51^ High-affinity binding is a common feature of cognate colicin DNase–Im protein complexes (*K*_d_ ≤ 10^− 14^ M), and the very tight binding between L18A–L19A–L37A and ColE7 (which rules out accurate determination of the *K*_d_ using direct titration experiments) is consistent with these observations.^52^ Such high-affinity binding is also consistent with the full protection against ColE7 observed *in vivo*.^52^ Indicative of binding-induced folding, far-UV CD experiments revealed an increase in the helical content of L18A–L19A–L37A upon addition of the ColE7 DNase domain (Fig. 6b). The most compelling evidence for binding-induced folding of L18A–L19A–L37A, however, was obtained using ^1^H–^15^N HSQC NMR, which showed a spectrum of the complex of unlabelled ColE7 DNase domain with ^15^N-labelled L18A–L19A–L37A (Fig. 6c), which is consistent with the spectrum of a folded colicin-bound immunity protein observed by Whittaker *et al*.^25^

### Residue-specific characterisation of the structural properties of unfolded Im7

**Fig. 7:**
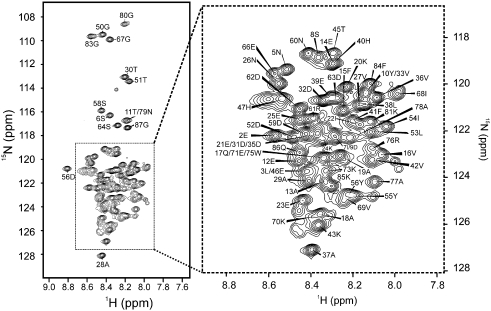
The 500-MHz ^1^H–^15^N HSQC spectrum of L18A–L19A–L37A. The spectrum was acquired in buffer A (90% ^1^H_2_O/10% ^2^H_2_O) with 0.2 M Na_2_SO_4_ at 10 °C. Sequential NOEs are observed for NH groups of *i* and *i* ± 1 residues for stretches of the protein involving residues 8–17, 18–37, 41–45, 51–52, 54–55, 60–63 and 67–69; however, no longer-range NOEs were observed in the three-dimensional HSQC-NOESY-HSQC experiment. The expanded region shows the assignments in crowded regions more clearly.

To determine the nature of residual structure in L18A–L19A–L37A and to characterise its dynamic properties, we obtained ^1^H–^15^N HSQC spectra in buffer A with 0.2 M Na_2_SO_4_, pH 7.0, at 10 °C (Fig. 7). Under these conditions, line broadening of the resonances is reduced compared with that observed in 0.4 M Na_2_SO_4_ (Fig. 4f). Although the folding pathway of Im7 has been shown to be independent of the concentration of Na_2_SO_4_,^39^ these conditions were chosen to permit assignment in conditions that most closely emulate previous folding studies.^24,26–28^ Using ^13^C/^15^N-labelled protein, we assigned 82 of 83 non-proline residues of L18A–L19A–L37A in the ^1^H–^15^N HSQC spectrum of the protein (Fig. 7). Assignments for 81 CO, 84 C^α^, 67 C^β^ and 66 H^α^ resonances of the variant were also obtained [Biological Magnetic Resonance Data Bank (BMRB) entry 17513]. The chemical shifts were analysed by comparison with reference random-coil chemical shift values to ascertain whether helical structure persists in the unfolded variant.^53,54^ This analysis revealed that no regions with regular secondary structure are present in the unfolded state (Fig. S1), consistent with analysis of this variant using far-UV CD (Fig. 3a). However, some small, systematic C^α^ chemical shift deviations are observed in regions of the protein corresponding to the native helices I and IV, suggesting that the unfolded ensemble could sample α-helical conformations transiently in these regions.

**Fig. 8:**
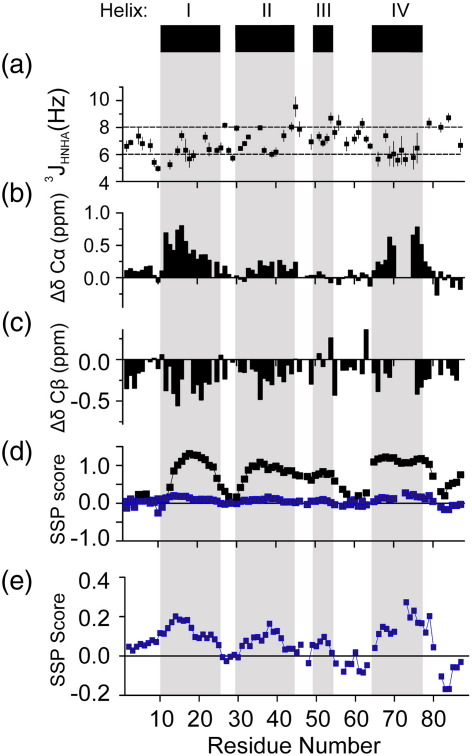
NMR analysis of the residual secondary structure in L18A–L19A–L37A. (a) ^3^J_HNHA_ coupling constants measured in buffer A (90% ^1^H_2_O/10% ^2^H_2_O) with 0.2 M Na_2_SO_4_ at 10 °C. Error bars reflect the errors in determining the peak heights estimated from the spectral noise levels. Horizontal lines mark the upper and lower boundaries for residues lying in extended (≥ 8 Hz) or helical regions (≤ 6 Hz).^55,56^ The positions of the helices in the native protein are indicated by the black bars above the plots with secondary structure taken from the X-ray structure of wild-type Im7 (PDB code: 1AYI^33^). Chemical shift differences between L18A–L19A–L37A in 0 M and 6 M urea for (b) C^α^ and (c) C^β^ resonances measured in buffer A (90% ^1^H_2_O/10% ^2^H_2_O) with 0.2 M Na_2_SO_4_ at 10 °C. The values collected in 6 M urea have been subtracted from those collected in the absence of urea. (d) SSP analysis of L18A–L19A–L37A in buffer A with 0.2 M Na_2_SO_4_ at 10 °C (blue) and wild-type Im7 (black) in buffer A. SSP analysis was performed using the published chemical shifts (BMRB entry: 7188) for wild-type Im7.^29^ (e) The SSP analysis for L18A–L19A–L37A in buffer A with 0.2 M Na_2_SO_4_ at 10 °C is shown expanded to highlight regions with significant, nonzero SSP scores.

To obtain further evidence of whether secondary structure persists within the unfolded ensemble, we measured ^3^J_HNHA_ coupling constants for L18A–L19A–L37A in 0.2 M Na_2_SO_4_, pH 7.0, at 10 °C. A total of 56 ^3^J_HNHA_ coupling constants could be measured, with values that range from 4.96 to 9.54 Hz (Fig. 8a). Of the 56 ^3^J_HNHA_ coupling constants, 39 are less than 8 Hz (the minimum value expected for ordered β-sheet structure) and 10 are less than 6 Hz (the maximum value expected for α-helical structure).^55^ The ^3^J_HNHA_ coupling constants also indicate a protein that lacks persistent secondary structure. Interestingly, the 10 residues with ^3^J_HNHA_ values that do fall below 6 Hz are found in regions of the sequence corresponding to the native helices I and IV, suggestive of an overall preference towards helical conformations in these regions (Fig. 8a).

The chemical shifts of L18A–L19A–L37A in 6 M urea were also obtained and used as a reference to compare with chemical shifts measured in 0 M urea. Using ^13^C/^15^N-labelled protein, we assigned 82 of 87 residues in the ^1^H–^15^N HSQC spectrum of L18A–L19A–L37A in 0.2 M Na_2_SO_4_ and 6 M urea at 10 °C (Fig. S2). Assignments for 82 CO, 82 C^α^ and 76 C^β^ resonances were also obtained (BMRB entry 17513). The C^α^ and C^β^ chemical shifts of L18A–L19A–L37A in buffer A with 0 M urea and 0.2 M Na_2_SO_4_ were then compared with the chemical shifts obtained for the same protein in buffer A with 6 M urea and 0.2 M Na_2_SO_4_ (Fig. 8b and c). The C^α^ secondary shifts are extremely sensitive to secondary structure and exhibit downfield-shifted (positive) values in α-helical regions.^57^ Using this analysis, we observed positive C^α^ secondary shifts for L18A–L19A–L37A in regions corresponding to the native helices I, II and IV (Fig. 8b). The C^β^ secondary shifts are also sensitive to secondary structure.^57^ Interestingly, the C^β^ secondary shifts of L18A–L19A–L37A are generally negative throughout the protein sequence (Fig. 8c). Although C^β^ chemical shifts are less sensitive to α-helix formation,^58^ the generally upfield-shifted C^β^ values are consistent with an overall increase in α-helical propensity upon dilution of the protein from denaturant (Fig. 8c).^57^ Although there are clear deviations in the chemical shifts of L18A–L19A–L37A in 0 M and 6 M urea, the C^α^ values all lie within 1 ppm of each other. By contrast, helix formation in native proteins generally results in C^α^ chemical shifts that change by ∼ 2.6 ppm.^57^ The small chemical shift differences of L18A–L19A–L37A in 0 M and 6 M urea indicate a significant, but weak, tendency towards helical structure in regions corresponding to the native helices I, II and IV in the absence of denaturant.

Next, the secondary structure propensity (SSP) scores were determined for L18A–L19A–L37A in 0 M urea using C^α^ and C^β^ chemical shifts. These values were then compared with those determined using previously published C^α^ and C^β^ chemical shifts for native wild-type Im7 in 0 M urea (BMRB entry 7188)^29^ (Fig. 8d and e).^59^ The SSP algorithm is designed to highlight conformational tendencies within an ensemble of unfolded or partially structured states.^59^ An SSP score of 1 indicates 100% α-helix propensity, whereas a score of − 1 indicates that the residue of interest has a 100% propensity to be in a β-strand conformation.^59^ The SSP scores for native, wild-type Im7 accurately predict the regions that are known to be α-helical in the native structure (Fig. 8d).^33^ By contrast, the SSP scores for L18A–L19A–L37A are much smaller, indicating an overall lack of persistent secondary structure. Nonetheless, there are inclinations towards helical conformations. The average SSP scores for regions that correspond to the native helices in L18A–L19A–L37A are shown in Table 2. These values support the previous results, demonstrating that helices I and IV have the highest helical propensity in the unfolded state. Notably, the SSP score indicates some helical tendency in helix III, which is not formed until very late in folding. However, the 4.3% helical propensity predicted by SSP is very small in comparison to the values obtained for helices I and IV, and this propensity is not reflected in the ^3^J_HNHA_ coupling constants for residues for which their values could be determined (Fig. 8a). Estimates of helical propensity using the prediction algorithm AGADIR^60^ and studies of synthetic peptides with sequences equivalent to the four helical regions of native Im7 also predict a weak helical propensity for these regions of the protein sequence that correlate with the SSP scores measured for L18A–L19A–L37A in 0 M urea (Table 2).^28^ These results suggest that the helical structure present in the unfolded state of Im7 reflects the natural helical propensity of the polypeptide sequence, rather than additional helical structure that may be induced by the collapse of the polypeptide chain in the absence of chaotrope.

### Polypeptide chain dynamics of unfolded Im7

**Fig. 9:**
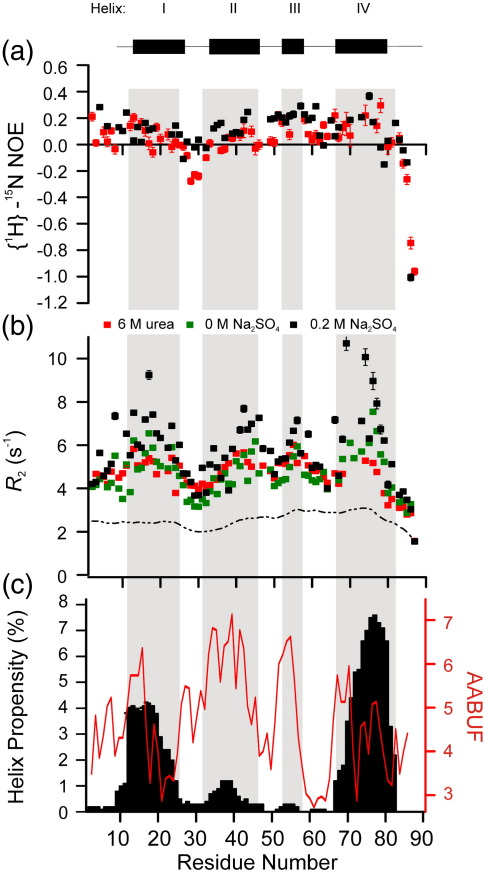
Backbone ^15^N relaxation rates and {^1^H}–^15^N NOEs of L18A–L19A–L37A measured in 0 M and 6 M urea. (a) {^1^H}–^15^N NOEs of the L18A–L19A–L37A in buffer A (90% ^1^H_2_O/10% ^2^H_2_O) with 0.2 M Na_2_SO_4_ and 0 M urea (black), or 6 M urea (red). (b) ^15^N transverse relaxation rates (50.66 MHz) in 0 M Na_2_SO_4_ (green), 0.2 M Na_2_SO_4_ (black) or 6 M urea with 0.2 M Na_2_SO_4_ (red). All data were acquired in buffer A (90% ^1^H_2_O/10% ^2^H_2_O) at 10 °C. Data are plotted against residue number, with native helices indicated by the grey-shaded regions. The broken line in (b) represents the intrinsic ^15^N *R*_2_ relaxation of a polypeptide chain with the sequence of L18A–L19A–L37A Im7 [determined using Eq. (8)]. Errors are shown but are smaller than the symbols for many of the data points. (c) Plots of AABUF (red) and the helix propensities (black) for L18A–L19A–L37A. AABUF values^61^ were calculated with the ExPASy tool ProtScale (http://www.us.expasy.org/tools/protscale.html) and normalised from 0 to 10. Helix propensities were calculated with AGADIR (pH 7.0, 10 °C).^60^

Backbone dynamics of L18A–L19A–L37A were investigated by measuring backbone transverse relaxation (^15^N *R*_2_) rates and {^1^H}–^15^N heteronuclear steady-state nuclear Overhauser enhancements (NOEs) (Fig. 9). {^1^H}–^15^N NOE data, determined for 62 residues, have values ranging from − 0.96 to 0.29 (Fig. 9a). The values indicate flexibility throughout the protein, as all {^1^H}–^15^N NOEs are below the average value (+ 0.78) expected for the amide groups of a rigid globular protein that is tumbling isotropically.^62^ The {^1^H}–^15^N NOEs measured in 6 M urea are very similar to those in 0 M urea (Fig. 9a), indicating that urea is not significantly modulating backbone dynamics that can be detected by this experiment (i.e., motion occurring on the picosecond-to-nanosecond timescale).^63^ Na_2_SO_4_ modulates line broadening in the ^1^H–^15^N HSQC spectrum of L18A–L19A–L37A (Fig. 4e and f). Therefore, the ^15^N *R*_2_ relaxation rates were measured in both 0 M and 0.2 M Na_2_SO_4_.^15^N *R*_2_ relaxation rates were measured for 75 out of 87 residues for this variant in 0 M Na_2_SO_4_/0 M urea, 76 out of the 87 residues of L18A–L19A–L37A in 0.2 M Na_2_SO_4_/0 M urea and 62 out of the 87 residues in 0.2 M Na_2_SO_4_/6 M urea (Fig. 9b). Analysis of the *R*_2_ rates reveals the presence of significant nonrandom structure in L18A–L19A–L37A indicated by increased *R*_2_ rates, relative to the intrinsic *R*_2_ values [estimated using Eq. (8)]. Interestingly, there is a small increase in the *R*_2_ rates of L18A–L19A–L37A in 0 M Na_2_SO_4_/0 M urea compared with the values obtained in 6 M urea, especially in residues located in helices I and IV (Fig. 9b). This could reflect decreased conformational flexibility in the unfolded state or an increase in the exchange between different species in the absence of the chaotrope. The *R*_2_ rates increase further in the presence of 0.2 M Na_2_SO_4_, consistent with further, now significant, conformational exchange between different species. Nonetheless, the clusters of residues with increased *R*_2_ rates in 0 M Na_2_SO_4_/0 M urea lie in regions that are helical in the native protein and correspond well with the average area buried upon folding (AABUF) (Fig. 9c). The AABUF is proportional to the hydrophobic contribution of a residue to the conformational free energy of a protein.^61^ Previous results have shown that the extent of residual secondary structure and AABUF correlate with sequence-dependent dynamic variations due to cluster formation in other denatured and unfolded states, such as acid-unfolded apomyoglobin,^64^ the Δ131Δ fragment of staphylococcal nuclease^65^ and the lambda repressor denatured state.^20^ This correlation for L18A–L19A–L37A suggests that hydrophobic interactions may be responsible for the deviations in *R*_2_ rates from those estimated from a random-coil model (Fig. 9b). The pattern of *R*_2_ rates of L18A–L19A–L37A in 0 M Na_2_SO_4_/0 M urea closely resembles the previously reported *R*_2_ rates of wild-type Im7 in 6 M urea, as well as those of L18A–L19A–L37A in 6 M urea (Fig. 9b).^29^ Four clusters (centred at L18, V42, Y56 and K73, named clusters I, II, III and IV) with increased *R*_2_ rates were previously interpreted to indicate hydrophobic clustering within aliphatic/aromatic-rich regions of the Im7 sequence that encompass the helix-forming regions of the protein (Fig. 9c).^29^ These results suggest that clustering of hydrophobic regions in the unfolded state of L18A–L19A–L37A is preserved even when the protein is dissolved in 6 M urea.

To determine whether the four hydrophobic clusters observed in L18A–L19A–L37A form long-range interactions, we created the amino acid substitutions F15A and F41A in L18A–L19A–L37A and we examined the effect of these substitutions on the dynamics of the entire polypeptide chain using ^15^N *R*_2_ relaxation measurements. F15A was previously chosen as probe for long-range interactions within the urea-unfolded state of Im7, since this is the largest hydrophobic residue in cluster I. Should the clusters interact, a significant change in the dynamic properties of clusters II, III and IV would be expected to occur in this variant.^29^ Similarly, F41A was chosen to probe for long-range interactions made by cluster II. Resonances of these variants were assigned by comparison with the spectrum of ^1^H–^15^N HSQC of L18A–L19A–L37A, and backbone ^15^N *R*_2_ relaxation rates were determined for 69 residues of the variant F15A–L18A–L19A–L37A and 58 residues of the variant L18A–L19A–L37A–F41A (Fig. S3a and b). Chemical shift changes for resonances surrounding the hydrophobic amino acid substitution sites were observed, resulting in residues 13–16 in the F15A–L18A–L19A–L37A variant and residues 39–45 in the L18A–L19A–L37A–F41A variant not being assigned (Fig. S3a and b). Nonetheless, chemical shift differences for the residues that could be assigned in each of the two variants were small compared with those of L18A–L19A–L37A, with F15A–L18A–L19A–L37A exhibiting average chemical shift changes of Δδ_N_ = 0.16 ± 0.14 ppm and Δδ_H_ = 0.03 ± 0.03 ppm, while L18A–L19A–L37A–F41A exhibits average chemical shift differences of Δδ_N_ = 0.06 ± 0.06 ppm and Δδ_H_ = 0.01 ± 0.02 ppm. Similarly, no significant change in *R*_2_ relaxation rates was observed in regions distant from the amino acid substitution site, while small deviations in *R*_2_ values close to the site of substitution were observed (Fig. S3). Together, the data suggest that the conformational properties of these variants are very similar to those of L18A–L19A–L37A and indicate that stabilising, intercluster hydrophobic interactions do not persist in the unfolded state of Im7 in the absence of urea. Instead, local clustering and hydrophobic residues are consistent with the chemical shift changes and deviations in *R*_2_ values close to the substitution site. Local hydrophobic clustering rather than long-range hydrophobic interactions were also observed for the wild-type Im7 in 6 M urea.^29^ Taken together, the data suggest that L18A–L19A–L37A is a dynamic ensemble of species that contains four clusters of locally interacting residues that lack persistent long-range hydrophobic interactions between the clusters but retain a small, overall preference for α-helical secondary structure.

## Discussion

### The unfolded state of Im7 is collapsed but lacks persistent secondary structure

The results above demonstrate that the sequence substitutions L18A, L19A and L37A destabilise Im7 significantly such that the unfolded state is predominantly populated under ambient conditions. The isodichroic point observed in the far-UV CD spectrum of all three variants in Fig. 3a indicates that all three destabilised variants are in conformational exchange between the unfolded state of Im7 and a more structured species. In addition, the similarity between the ^1^H–^15^N HSQC spectrum of the L18A–L19A and the L18A–L19A–L37A variant in the absence of Na_2_SO_4_ provides compelling evidence, that despite the differences in amino acid substitutions, both the proteins populate comparable unfolded states. Furthermore, the ability of L18A–L19A–L37A to populate a native-like conformation has been demonstrated, indicating that the amino acid substitutions introduced into the protein have not disrupted the sequence information required to adopt the native fold. Together, these results suggest that the L18A–L19A–L37A variant is an excellent mimic of the unfolded state of Im7 in ambient conditions.

Measurements of the properties of this species using far- and near-UV CD, fluorescence and NMR indicate that this protein lacks significant secondary structure and persistent tertiary interactions. Nonetheless, the ensemble possesses an overall residual helicity of approximately 6–9% as measured by far-UV CD spectroscopy and NMR (overall SSP score) that results from the inherent properties of the Im7 sequence (local helical propensity and hydrophobicity). The unfolded state created here is approximately 30% more compact than its counterpart in 6 M urea, qualitatively in agreement with observations made by smFRET studies that revealed compaction of the unfolded state in the absence of urea.^24^ The overall low helicity of the unfolded ensemble of L18A–L19A–L37A is consistent with measurements of the helical propensities of synthetic peptides with sequences corresponding to the individual helices of Im7 and using the secondary-structure prediction algorithm AGADIR.^28,60^ Considering all of the experimental data obtained, the unfolded state of Im7 is envisaged to be collapsed, to lack persistent long-range interactions and to possess only inklings of helical structure.

### Implications for the folding of Im7

Recent extensive simulations of rapidly folding proteins suggest that there is a strong correlation between helical propensity within the unfolded state and the order of formation of local native-like structure along the folding transition path.^66^ Additionally, the formation of only a subset of key long-range native contacts in the unfolded state may be sufficient to establish a native-like topology and stabilize the transiently formed secondary structure.^66^ Despite lacking persistent interactions, residual structure in the unfolded state of Im7 could contribute to the determination of the rate and/or mechanism of folding to the native state. In this context, it is interesting to note that all four regions corresponding to helices in native Im7 populate helical conformations (Table 2). It is somewhat surprising that helix III shows a tendency towards α-helical conformations given this is the last helix to form during folding and has little, if any, predicted helical propensity (Table 2). Studies on the folding mechanism of Im7 have shown that helices I and II form crucial interactions within the first transition state (TS1) for folding (Fig. S1), specifically involving residues L18, L19 and L37.^27^ Despite substituting these three leucine residue with alanine in L18A–L19A–L37A, the sequence is still able to fold to a native conformation provided that suitable stabilising conditions are formed (herein, provided by binding to ColE7). Conformational exchange observed in the presence of Na_2_SO_4_ suggests that L18A–L19A–L37A is able to fold into the intermediate state, albeit transiently. Previous Φ-value analysis has shown that as Im7 folds from TS1 to the I-state, helix IV forms nonnative interactions with helices I and II, precluding the docking of helix III.^27^ Helix IV is the most populated helix within L18A–L19A–L37A and its pre-organisation may contribute to the folding mechanism. Together, these data confirm the validity of using L18A–L19A–L37A as a mimic of the unfolded state of the wild-type protein, since the sequence is able to fold to both the on-pathway intermediate and native states.

Comparison of the results presented here, along with previous studies of urea-denatured Im7,^29^ reveals that the unfolded states of Im7 in 0 M and 6 M urea have remarkably similar structural properties, since neither ensemble has significant secondary structure, both contain four elements of local hydrophobic clusters and neither contains persistent tertiary interactions. Nonetheless, significant changes in the unfolded state of Im7 occur upon dilution from denaturant. Firstly, the unfolded state becomes more compact in the absence of urea. Additionally, an overall increase in helicity, particularly in helices I and IV, occurs when the chaotrope is removed, which could drive, or be driven by, the concomitant collapse.^67^ In the case of the wild-type unfolded sequence, the formation of stabilising long-range interactions, particularly involving side chains in helices I and II, would trigger folding.^26,27^ Transient long-range interactions within the unfolded state of Im7 have not yet been probed and further investigation will be required to reveal whether such interactions prime the ensemble to fold by transiently sampling the persistent interactions that are formed in TS1. Persistent interactions define TS1 and, thereby, mark the first stable interactions that commit the sequence of Im7 to fold to a compact globular structure.

### Comparison with residual structure in other model proteins

Compaction of the polypeptide chain under nondenaturing conditions has been observed for many protein sequences placed under conditions that do not permit folding.^10,19,68,69^ For example, a radius of gyration or hydration that is approximately 1.3–1.5 times that of the folded state has been reported for the *Drosophila* drk N-terminal (drkN) domain,^10^ the N-terminal (NTL9) and the C-terminal domain (CTL9) of ribosomal protein L9^19,68^ and the Δ131Δ fragment of staphylococcal nuclease.^65,69^ These measurements of compaction are comparable to the observations made here for L18A–L19A–L37A, for which the *R*_h_ is 1.4 times larger than that of native wild-type Im7. Interestingly, Anil *et al.* demonstrated that the same unfolded sequence could have markedly different levels of secondary structure yet share the same *R*_h_ values, suggesting that compaction should not be considered diagnostic of residual structure.^19^ Furthermore, Kohn *et al.* have shown that even a random unfolded polypeptide chain exhibits compaction compared with its corresponding urea-denatured state, indicating that compaction of the unfolded state need not be mediated by sequence-dependent interactions.^70^ Collapse is likely mediated by hydrophobic and electrostatic interactions that could be driven by a combination of nonspecific structural properties of the protein and also specific interactions that could be important for folding.^71^ Further experiments, for example, using paramagnetic relaxation enhancements and relaxation dispersion NMR methods combined with detailed simulation methods, such as those studies conducted in the Forman-Kay,^10,72^ Vendruscolo,^21^ Poulsen^73^ and Blackledge^74^ and co-workers, will be required to define the origins of the residual structure observed in the unfolded state of L18A–L19A–L37A in more detail.

Although it is difficult to classify and compare the extent of residual structure within unfolded states of different protein, it is becoming clear that these ensembles form a broad range of structures.^76^ The extent of observed residual structure ranges from highly disordered states (e.g., chymotrypsin inhibitor 2^76^) to denatured states that possess significant residual structure and persistent tertiary contacts in nondenaturing conditions, such that they can be classed as folding intermediates (e.g., engrailed homeodomain^18^). The results presented here suggest that the unfolded state of Im7 lacks persistent structure and is nearer the “disordered” end of this spectrum of unfolded protein conformers. The lack of persistent helical structure in the unfolded state of Im7 is consistent with a nucleation–condensation mechanism for the intermediate formed from the unfolded state that defines the first stage of Im7 folding. In members of the homeodomain family, the extent of residual helical structure in the denatured state corresponds to a spectrum of folding mechanisms from diffusion–collision for highly structured denatured proteins to nucleation–condensation in proteins that lack significant helical propensity in their denatured state.^77^ Hydrophobic interactions have also been implicated as important sites for initiation of protein folding.^5^ The results presented here add to the emerging understanding of residual structure in the unfolded ensembles of α-helical proteins. A combination of secondary structure propensity and hydrophobic interactions determines the nature of subsequent folding steps taken and ultimately leads to the malleability observed in the transition states populated during folding.^77^ In the case of Im7, both hydrophobic clustering in specific regions of the polypeptide chain and local helical propensity define the starting point of folding, which ultimately leads to an efficient search to folded species that are required for colicin binding and cell survival.^36,37^

## Materials and Methods

### Expression, purification and mutagenesis

All variants were created from wild-type Im7 containing an N-terminal hexahistidine tag.^30^ Mutants were created using the Stratagene QuikChange Site-Directed Mutagenesis Kit following the manufacturer's instructions, except that the incubation time with DpnI was increased from 1 h to overnight. Genes were sequenced to confirm that mutagenesis had been successful. Proteins were overexpressed in *E. coli* cells and purified as described previously,^38^ with the exception that anion-exchange chromatography was carried out using Source 15Q resin (GE Healthcare). Protein was loaded onto the column in 50 mM sodium phosphate (pH 6.0), the column was washed with 2 column volumes of this buffer and protein was eluted with a gradient of 0–0.65 M NaCl. ^15^N/^13^C-labelled proteins were expressed in BL21(DE3) cells and grown in ^15^N/^13^C-enriched medium containing 1 g/L of ^15^NH_4_Cl and 2 g/L of uniformly labelled ^13^C-glucose. Protein was shown to be   >    95% pure using SDS-PAGE and of the expected mass (determined using electrospray ionisation mass spectrometry). Unless indicated otherwise, all proteins were dissolved in buffer A containing 50 mM sodium phosphate and 1 mM EDTA (pH 7.0).

### Circular dichroism

Data were collected on a Jasco J715 CD spectropolarimeter (Great Dunmow, Essex, UK). The CD signal for measurements in the far-UV region (200–260 nm) was recorded in a  1-mm-path-length cell with a protein concentration of 0.2 mg/ml. Far-UV CD spectra were recorded using a 1- nm bandwidth, a 1- nm resolution, a 20- nm/min scan speed and a response time of 8 s, averaging 5 scans. Measurements in the near-UV CD spectrum (250–350 nm) were recorded at a protein concentration of 1.5 mg/ml using a  1-cm-path-length cell, with a 1- nm bandwidth, a 0.5- nm resolution, a 10- nm/min scan speed and a response time of 4 s, averaging over 12 scans. The measured mean residue ellipticity was used to estimate helical content, using Eq. (1). The scaling factor 50 is included as 50% of the residues in wild-type Im7 are in a helical conformation, as defined in the Protein Data Bank (PDB) entry 1AYI.^33^

(1) $$\text{\%}\text{ Helical Content}=\left(\frac{{\text{MRE}}_{\text{222}\;\text{nm}}^{\text{variant}}}{{\text{MRE}}_{\text{222}\;\text{nm}}^{\text{Im7}}}\right)\times 50$$

### Equilibrium denaturation curves

Data were collected on a Photon Technology International fluorimeter (Ford, West Sussex, UK), with a  1-cm-path-length cuvette or a Jasco J715 CD spectropolarimeter with a  1-mm-path-length cuvette. Solutions of protein containing 0–8 M urea in 0.2- M increments were used, with a final protein concentration of 0.1 mg/ml for fluorescence samples and 0.2 mg/ml for far-UV CD samples. All samples were incubated overnight at 10 °C before measurements were taken. The CD signal at 222 nm was measured using a bandwidth of 1 nm, a response time of 1 s and the average signal taken over 1 min. Data for the wild-type protein were fitted to a model describing a two-state transition:

(2) $$\text{Signal}=\frac{\left(\left(a\left[\text{urea}\right]+b\right){\text{exp}}^{\left(\Delta G_{\text{UF}}^{\circ }-M_{\text{UF}}\left[\text{urea}\right]\right)/RT}+\left(c\left[\text{urea}\right]+d\right)\right)}{\left(1+{\text{exp}}^{\left(\Delta G_{\text{UF}}^{\circ }-M_{\text{UF}}\left[\text{urea}\right]\right)/RT}\right)}$$

where Δ*G*°_UF_ (kJ mol^− 1^) is the equilibrium stability, *M*_UF_ is the equilibrium *m*-value, *a* and *c* represent the denaturant dependence of the folded and unfolded signal intensities, respectively, and *b* and *d* are the signal intensities of the folded and unfolded states, respectively, in the absence of denaturant. The fluorescence intensity was an insensitive probe of protein folding for the variants and therefore fluorescence emission spectra were monitored, using an excitation wavelength of 280 nm. Fluorescence emission was recorded from 300 to 400 nm in  1-nm increments, integrating for 1 s at each wavelength.

### Analytical ultracentrifugation

Sedimentation velocity experiments were carried out with a Beckman Optima XL-I analytical ultracentrifuge (Beckman, Palo Alto, CA) using an An-50 Ti or An-60 Ti rotor at 10 °C, 50,000 rpm, and using conventional aluminum double-sector centerpieces with sapphire windows. Samples were prepared by dissolving lyophilised protein in buffer A followed by overnight dialysis against this buffer. The dialysate was used as a buffer blank solution. Radial absorbance scans (λ = 280 or 296 nm) were collected at 300-s intervals. The sedimentation coefficients (*S*) were analysed in terms of a size distribution function *c*(*S*) using the program SEDFIT.^78^ Experimentally determined sedimentation coefficients were corrected to standard conditions (20 °C) in water to give corrected sedimentation coefficient (*S*_20,w_) values using the solvent density (ρ) and protein partial specific volumes (*v̅*), which were calculated using SEDNTERP software†.^79^ *S*_20,w_ values were used according to the Svedberg equation to determine the translational friction coefficient (*f*) of the protein using Eq. (3), where *M* is the molecular weight of the protein, *v̄* and ρ_20,w_ are the partial specific volume of the protein and solvent density, respectively, calculated under standard conditions and *N*_A_ is Avogadro's number. Sedimentation coefficients are reported in Svedberg units (S), which correspond to 10^− 13^ s.^80^

(3) $$S_{20,\text{w}}=\frac{M\left(1-{\overset{―}{v}}_{20,\text{w}}\rho_{20,\text{w}}\right)}{N_{\text{A}}f}$$

Experimentally determined frictional coefficients were compared with the frictional coefficient that would be expected for a rigid, hydrated spherical molecule of equal volume (*f*_o_) determined using Eq. (4), based on the Stokes equation,^80^ where η is the viscosity of the solution and *R*_0_ is the radius of the sphere.

(4) $$f_{0}=6\pi \eta R_{0}$$

Frictional ratios (*f*/*f*_o_) provide a measure of the compaction of the protein compared with a perfect sphere.^80^ Since the frictional coefficient of the protein is proportional to the radius of hydration (*R*_h_), it has been used to determine an estimate of the *R*_h_ for the proteins studied, by comparison with the Stokes radius (*R*_0_) using Eq. (5).

(5) $$\frac{f}{f_{0}}\cdot R_{0}=R_{h}$$

### *In vivo* colicin assays

JM83 *E. coli* cells were transformed with a pTrc99a plasmid encoding the Im7 gene of interest and inoculated into 0.7% LB agar containing 50 μg/ml carbenicillin and 1 mM IPTG. This was poured onto a precast 1.5% LB agar plate, and once set, a serial dilution of E7 colicin was spotted onto the plate. This assay is described by Wallis *et al*.^48^

### *In vivo* LuxA assays

This assay makes use of an SOS-inducible chromosomal *lux* operon to detect DNA damage induced by ColE7 in reporter cells.^50^ *E. coli* DPD1718 contain a fusion of the *E. coli recA* promoter region with the *Photorhabdus luminescens luxCDABE* reporter integrated into the lacZ locus of *E. coli* DPD1692.^50^ *E. coli* DPD1718 were transformed with a pTrc99a plasmid encoding the Im7 gene of interest, and overnight cultures were grown in LB at 37 °C in  10-ml culture volumes. Cultures were then diluted 1:50 into a final volume of 100 ml of LB media and grown for 2 h at 37 °C in the presence of 30 μg/ml chloramphenicol and 50 μg/ml carbenicillin. We then added 1 mM IPTG to the cultures, and they were allowed to grow for another hour until an OD_600_ of 0.4–0.5 was reached within the culture flasks. The cells were then diluted 1:2 and 100 μl was added to a 96-well optical bottom microtitre plate (Corning®) pre-warmed to 37 °C. ColE7 (4 μl; final concentration, 4 nM) was then added. All assays were performed in a PerkinElmer EnVision microtitre plate reader. Induction of luminescence was followed over a period of 90 min, with readings taken every 600 s. Cell densities were monitored by measuring the OD at 492 nm. The gamma values were determined at the 50 -min time point according to Eq. (6), where *L*_sample_ is the luminescence value of the cell strain of interest corrected for cell density and *L*_control_ is the cell density corrected luminescence value of control cells that were not challenged with ColE7.

(6) $$\gamma =\frac{L_{\text{sample}}-L_{\text{control}}}{L_{\text{control}}}$$

### Isothermal titration calorimetry

Experiments were carried out using a VP-ITC (Microcal) calorimeter following the manufacturer's instructions. Briefly, lyophilised protein samples were extensively dialysed overnight at 4 °C in the assay buffer (50 mM MOPS at pH 7.0 containing 1 mM EDTA). The assay buffer and proteins were filtered (0.22 μM pore size) and diluted to the working concentration (10 μM ColE7 DNase domain and 120 μM L18A–L19A–L37A) and degassed immediately prior to use. Experiments were performed at 298 K with an initial injection of 5 μl followed by a series of injections of 7.5 μl. The resulting binding isotherm was fitted to a 1:1 binding model using the software provided by the manufacturer, although the high affinity of the complex prohibited accurate measurement of the *K*_d_ from this experiment.

### NMR spectroscopy

All NMR experiments were carried out at 10 °C using Varian Unity Inova spectrometers operating at ^1^H frequencies of 500, 600 and 750 MHz. Protein samples in the range of 0.2–0.5 mM were prepared in buffer with 10% D_2_O and in the presence of 0 M, 0.2 M or 0.4 M Na_2_SO_4_, or 0.2 M Na_2_SO_4_ and 6 M urea, as indicated. Gradient-enhanced ^1^H–^15^N HSQC spectra were acquired using 128 complex points and 16 scans per increment with spectral widths of 8511 Hz and 1800 Hz in the ^1^H and ^15^N dimensions, respectively. Watergate solvent suppression was used, and all NMR data were processed using NMRPipe and analysed in NMRView.^81,82^

Although the Im7 construct used has an additional histidine tag at the N-terminus, assignments have been labelled according to the corresponding residue number in the wild-type untagged sequence. Peak assignments of the L18A–L19A–L37A Im7 variant were determined in the presence of 0.2 M Na_2_SO_4_ and in the same buffer with the addition of 6 M urea. ^1^H chemical shifts were referenced directly to external DSS, and the ^13^C and ^15^N chemical shifts were referenced indirectly to DSS. ^15^N,^13^C-labelled proteins were used to obtain sequential connectivity of C^α^ and C^β^ resonances by performing HNCACB, CBCA(CO)NH, HN(CO)CA and HNCA experiments. The assignments of the L18A–L19A–L37A Im7 variant in the absence of urea were further confirmed by performing a  120-ms three-dimensional ^1^H–^15^N HSQC-NOESY (nuclear Overhauser enhancement spectroscopy)-HSQC experiment. An HNHA experiment was also performed to obtain H^α^ resonance assignments for L18A–L19A–L37A in the absence of urea. Chemical shift differences were calculated using the temperature, pH and sequence-corrected chemical shifts of Kjaergaard *et al*.^53,54^ SSP analysis was performed using C^α^ and C^β^ chemical shifts using the algorithm developed by Marsh *et al.* using the software provided‡.^59^

Homonuclear three-bond J_(HNHA)_ coupling constants were determined by an HNHA experiment on ^15^N-labelled L18A–L19A–L37A in buffer A (90% ^1^H_2_O/10% ^2^H_2_O) in the presence of 0.2 M Na_2_SO_4_ at 10 °C. A mixing time (2Δ) of 25.08 ms was used in the pulse sequence described by the method developed by Vuister and Bax.^83^ Coupling (J_NH_) constants can be determined by the diagonal (*I*_diag_) and cross-peak (*I*_cross_) intensities using Eq. (7), where Δ is the experimental delay in the pulse sequence.^83^ Errors were determined from the error in measuring the peak heights determined from the noise level of the spectra.

(7) $$\frac{I_{\text{cross}}}{I_{\text{diag}}}=-{\text{tan}}^{2}\left(2\pi {\text{J}}_{\text{NH}}\Delta \right)$$

Backbone ^15^N transverse relaxation measurements were carried out as previously described,^62^ for samples in buffer A. An estimate of the error was determined using duplicate points and spectral noise levels. Relaxation measurements were performed at 500 MHz using a series of 11 experiments with mixing times ranging from 1.60 to 191.6 ms. Backbone ^15^N *R*_2_ relaxation rates were modelled and estimated using the approach previously described in Le Duff *et al*.^29^ The per-residue sequence-dependent intrinsic *R*_2_ values due to polymer hydrodynamics were calculated from the radius of gyration of each residue and its neighbours modulated by an exponential decrease in persistence along the chain. Sequence-dependent relaxation rates in the absence of clustering were given by Eq. (8):

(8) $$R_{2i}=k_{R_{2i}}\cdot \sum_{J=1}^{N}\tau_{j}\cdot e^{\frac{-\left|i-j\right|}{\lambda_{j}}}$$

where $k_{R_{2i}}$ is an empirical scaling coefficient, *R*_2*i*_ is the intrinsic transverse relaxation rate of each residue, *N* is the number of residues and τ*_j_* and λ*_j_* are the intrinsic correlation time of each residue and the persistence length for segmental motion of the polypeptide, respectively. It was assumed that λ_*j*_ was 2 for motion deriving from alanine and glycine and 7 for all other residue types. The intrinsic correlation time for each residue, τ*_j_*, was calculated from Stokes law using Eq. (9):

(9) $$\tau_{j}=k_{\text{rg}}\cdot R_{\text{g}}^{3}$$

where *k*_rg_ is an arbitrary scaling factor that was amalgamated with $k_{R_{2i}}$ and *R*_*g*_ is the radius of gyration for each residue. Values of *R*_g_ were obtained from Levitt,^84^ via the database§ with the exception that the *R*_g_ for proline was increased from 1.25 Å to 2 Å to allow for the reduction of conformational space due to ring closure in the peptide, previously described in the literature.^85^

Heteronuclear NOEs were measured with the procedure and pulse sequences described by Farrow *et al*.^62^ Proton saturation was achieved with a 120° pulse applied every 5 ms over 3 s during the  5-s relaxation delay. NOE values were determined as the ratio of the average peak heights with and without proton saturation, with the uncertainties of the NOE values taken to be error in measuring the peak heights determined from the noise level of the spectra.

### Data bank accession number

Sequence-specific assignments for L18A–L19A–L37A in buffer A with 0.2 M Na_2_SO_4_ and 0 M or 6 M urea have been deposited in the BMRB as entry 17513.

## Figures and Tables

**Table 1:** Hydrodynamic properties of wild-type Im7 and the L18A–L19A–L37A variants at 10 °C

	*S*_20,w_^a^	*f*/*f*_o_	*R*_h_ (Å)	*C*^b^	MM_apparent_ (kDa)
Wild type^c^	1.45 ± 0.03	1.21 ± 0.02	17.8 ± 0.3	0.99	10.6 ± 0.3
L18A–L19A–L37A^d^	0.99 ± 0.18	1.79 ± 0.04	26.1 ± 0.6	0.28	13.3 ± 2.4
L18A–L19A–L37A^d^^,^^e^ (0.4 M Na_2_SO_4_)	0.90 ± 0.03	1.70 ± 0.05	24.6 ± 0.8	0.41	10.4 ± 0.2
L18A–L19A–L37A^d^^,^^f^ (6 M urea)	0.75 ± 0.04	2.33 ± 1.73	34.0 ± 1.7	< 0	—

a Given as 10^− 13^ s.

b Compaction factors calculated using the equation *C* = (*R*_D_^h^ − *R*_h_)/(*R*_D_^h^ − *R*_N_^h^) as described by Wilkins *et al.*^47^*R*_N_^h^ and *R*_D_^h^ are the predicted values of hydrodynamic radii for the native and fully denatured states, respectively, and *R*_h_ is the experimentally determined hydrodynamic radius.

c Error is standard deviation from three samples at 50 μM, 60 μM and 100 μM protein concentration.

d Error is standard deviation from three samples at 50 μM, 60 μM and 400 μM protein concentration.

e Sample in buffer A with 0.4 M Na_2_SO_4_.

f Sample in buffer A with 6 M urea.

**Table 2:** Helical propensity of the Im7 sequence

Helix	Residues^a^	Wild-type % helix^b^	Measured for isolated peptide^c^	SSP (L18A–L19A–L37A) (% helix)^d^
I	12–25	10.0	14.1	13.1
II	31–45	1.6	9.8	8.1
III	51–56	0.2	0	4.3
IV	65–79	5.4	11.2	16.3

a Native α-helical regions in wild-type Im7 (taken from 1AYI^37^).

b AGADIR^55^ prediction of the average helical content of wild-type Im7 for the region indicated at 10 °C, pH 7.0.

c Values quoted are from Knowling *et al*.^28^

d Average SSP^59^ score for this region of L18A–L19A–L37A, determined using C^α^ and C^β^ chemical shifts.

## Footnotes

† Available at http://www.rasmb.bbri.org

‡ http://pound.med.utoronto.ca/software.html

§ http://www.genome.jp/aaindex
