# Supplementary material for: Conformational Properties of the Unfolded State of Im7 in Nondenaturing Conditions
Source: J Mol Biol. 2012 Feb 17;416-318(2-12):300–18. doi: 10.1016/j.jmb.2011.12.041 (PMC3314952; doi:10.1016/j.jmb.2011.12.041)
Supplement: Supplementary file 1 — Supplementary materials [file mmc1.pdf]

Supplementary Information

Figure Legends

SI Figure 1

Chemical shift deviations from random coil values determined for L18A L19A L37A. Chemical shifts were obtained in buffer A (90 % ^1H_2O/10 % ^2H_2O) with 0.2 M Na_2SO_4 at 10 °C. The $\Delta\delta$ values for (from top to bottom) HN, N, CO, Hα, Cα and Cβ resonances display the deviations from the sequence-corrected random coil values reported by Kjaergaard *et al.*[48,49] The position of the helices in the native protein are indicated by the black bars above the plots, with secondary structure taken from the X-ray structure of native Im7 (PDB: 1AYI[36]).

SI Figure 2

The 500 MHz ^1H-^{15}N HSQC spectrum of L18A L19A L37A in 6 M urea. The spectrum was acquired in buffer A (90 % ^1H_2O/10 % ^2H_2O) with 0.2 M Na_2SO_4, 6 M urea and 1mM EDTA at 10 °C.

SI Figure 3

Transverse relaxation rates for hydrophobic substitution variants of L18A L19A L37A. The 500 MHz ^{15}N R_2 relaxation rates for (a) F15A L18A L19A L37A and (b) L18A L19A L37A F41A are plotted (red solid data points) against residue number with the relaxation rates of L18A L19A L37A plotted in each case (black solid data points). The solid black line in each plot depicts the theoretical rates calculated for a random coil with the sequence of L18A L19A L37A (Materials and Methods). Data in this figure were acquired in buffer A (90 % ^1H_2O/10 % ^2H_2O) with the addition of 0.2 M Na_2SO_4 at 10 °C. The position of the helices in

the native protein are indicated, with secondary structure taken from the X-ray structure of native Im7 (PDB: 1AY[36]).

SI Figure 1

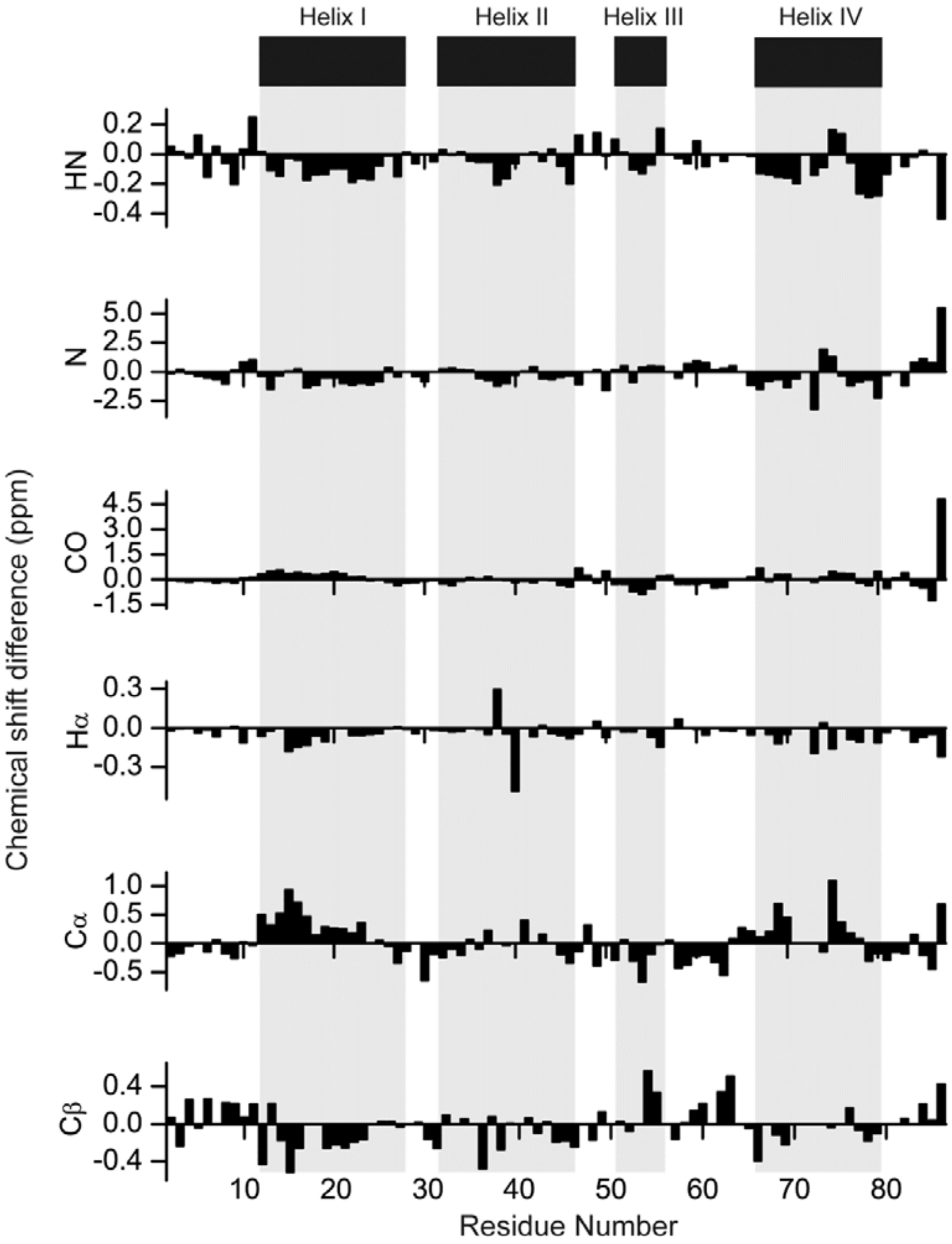

Helix I
Helix II
Helix III
Helix IV
HN
0.2
0.0
-0.2
-0.4
N
5.0
2.5
0.0
-2.5
CO
4.5
3.0
1.5
0.0
-1.5
Hα
0.3
0.0
-0.3
Cα
1.0
0.5
0.0
-0.5
Cβ
0.4
0.0
-0.4
Chemical shift difference (ppm)
10 20 30 40 50 60 70 80
Residue Number

SI Figure 2

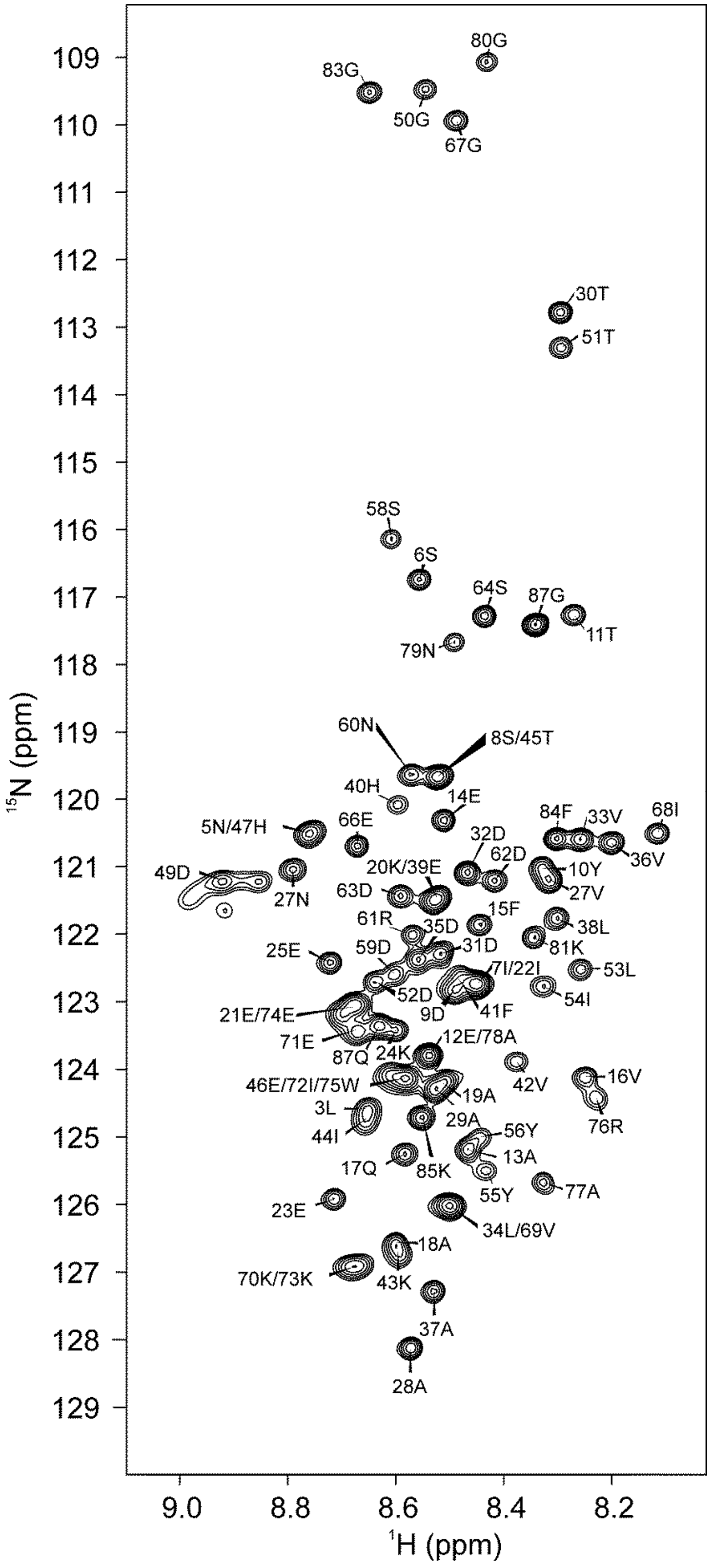
80G
83G
50G
67G
30T
51T
58S
6S
64S
87G
11T
79N
60N
8S/45T
40H
14E
84F
33V
68I
5N/47H
66E
32D
36V
62D
49D
20K/39E
10Y
27N
63D
27V
15F
61R
35D
38L
25E
59D
31D
81K
53L
7I/22I
52D
54I
21E/74E
9D
41F
71E
87Q
24K
12E/78A
46E/72I/75W
42V
16V
3L
19A
29A
44I
56Y
76R
13A
17Q
85K
55Y
77A
23E
34L/69V
18A
70K/73K
43K
37A
28A
109
110
111
112
113
114
115
116
117
118
119
120
121
122
123
124
125
126
127
128
129
¹⁵N (ppm)
9.0
8.8
8.6
8.4
8.2
¹H (ppm)

SI Figure 3

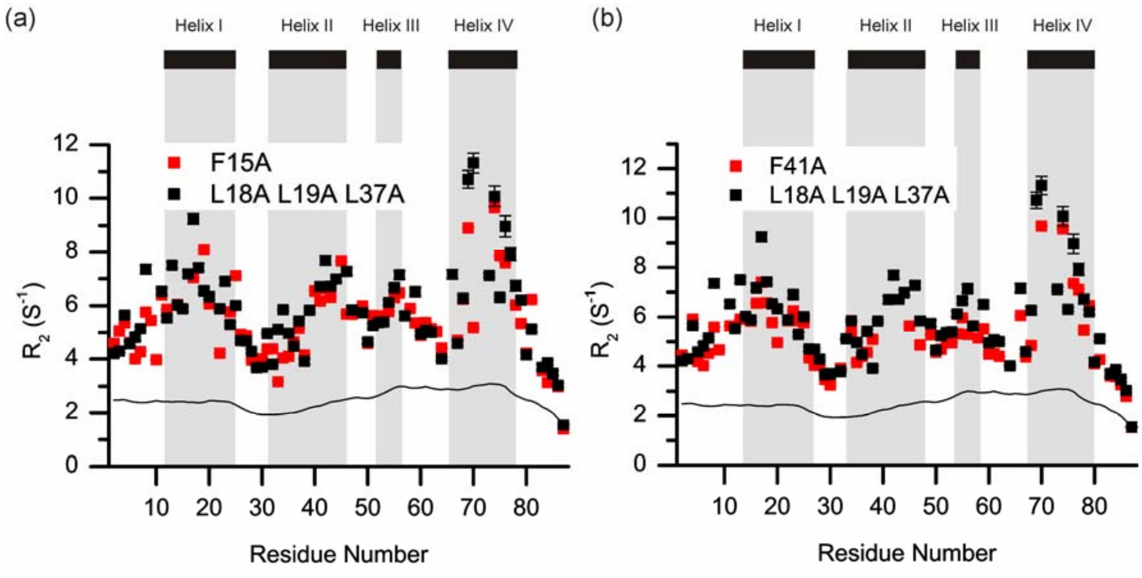
(a)
Helix I
Helix II
Helix III
Helix IV
F15A
L18A L19A L37A
R_2 (S^{-1})
Residue Number
(b)
Helix I
Helix II
Helix III
Helix IV
F41A
L18A L19A L37A
R_2 (S^{-1})
Residue Number
